# Supplementary material for: cATR Tracing Approach to Identify Individual Intermediary Neurons Based on Their Input and Output: A Proof-of-Concept Study Connecting Cerebellum and Central Hubs Implicated in Developmental Disorders
Source: Cells. 2022 Sep 24;11(19):2978. doi: 10.3390/cells11192978 (PMC9562212; doi:10.3390/cells11192978)
Supplement: Supplementary file 1 [file cells-11-02978-s001.zip › cells-1890867-supplementary.pdf]

Supplementary Material

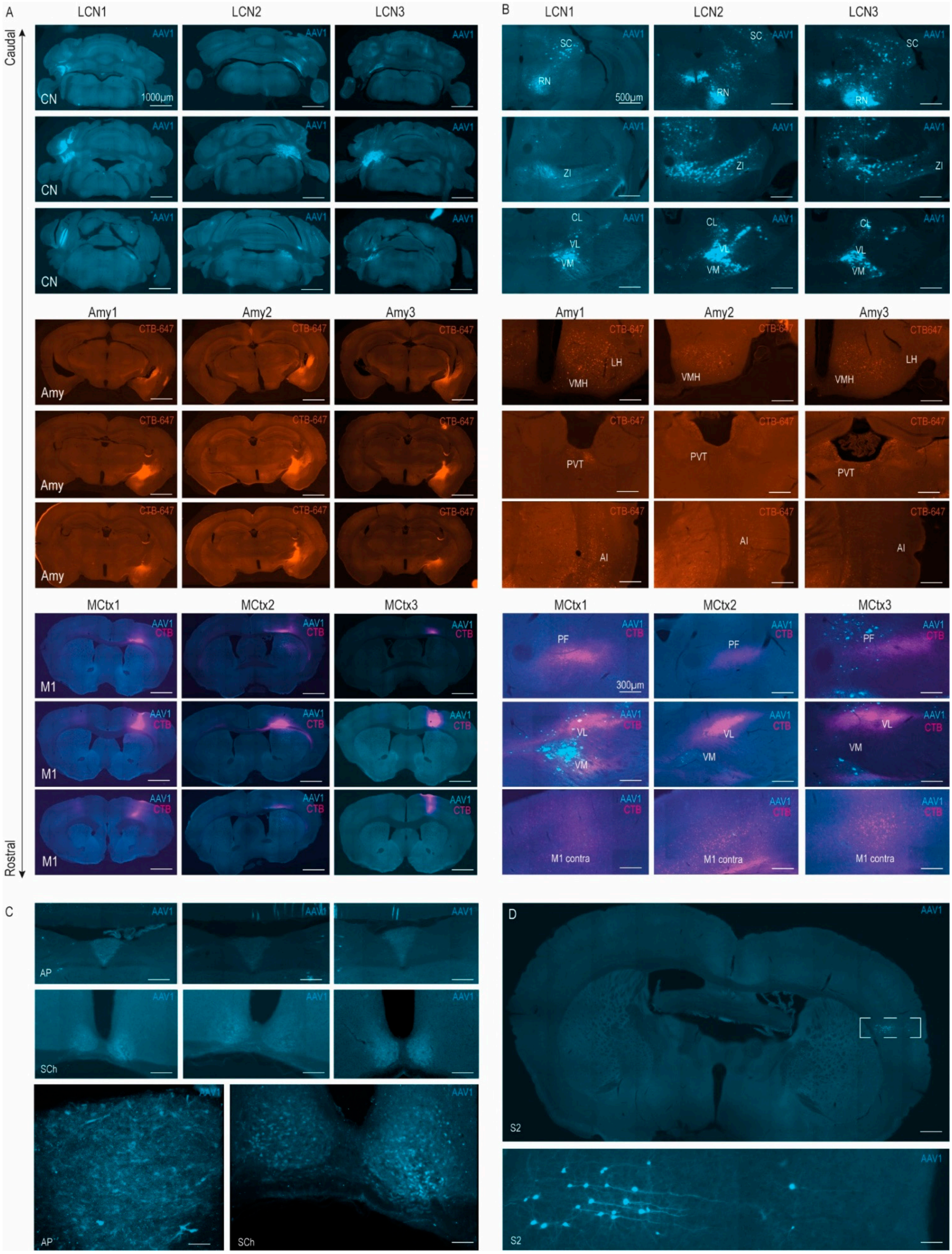

Supplementary Figure S1. Overview of all slices containing injection spots and examples of brain-wide labeling in LCN-M1/amygdala mice. (**A**) Overview of the center of injection spot and most caudal and rostral spread in each target area (LCN, amygdala and M1) for each mouse separately. (**B**) examples of anterograde and retrograde labeling in known targets for LCN, amygdala and M1 for those same mice. (**C**) overview pictures of putative AAV1 labeling in AP and SCh. (**Bottom**) confocal scan of AP, showing few AAV1+ cells, and dense labeling of cells in SCh. (**D**) Overview of AAV1+ cells in S2 (**top**) and magnification of area in top (**bottom**). Abbreviations: AI, anterior insula; Amy, amygdala; AP, area postrema; CL, centrolateral thalamic nucleus; CN, MCN-INT-LCN injections; M1, primary motor cortex; PF, parafascicular nucleus; PVT, paraventricular thalamic nucleus; RN, red nucleus; SCh, suprachiasmatic nucleus; SC, superior colliculus; S2, second ssomatosensory cortex; VM, ventromedial thalamic nucleus; VMH, ventromedial hypothalamic nucleus; VL, ventrolateral thalamic nucleus; VTA, ventral tegmental area; ZI, zona incerta.

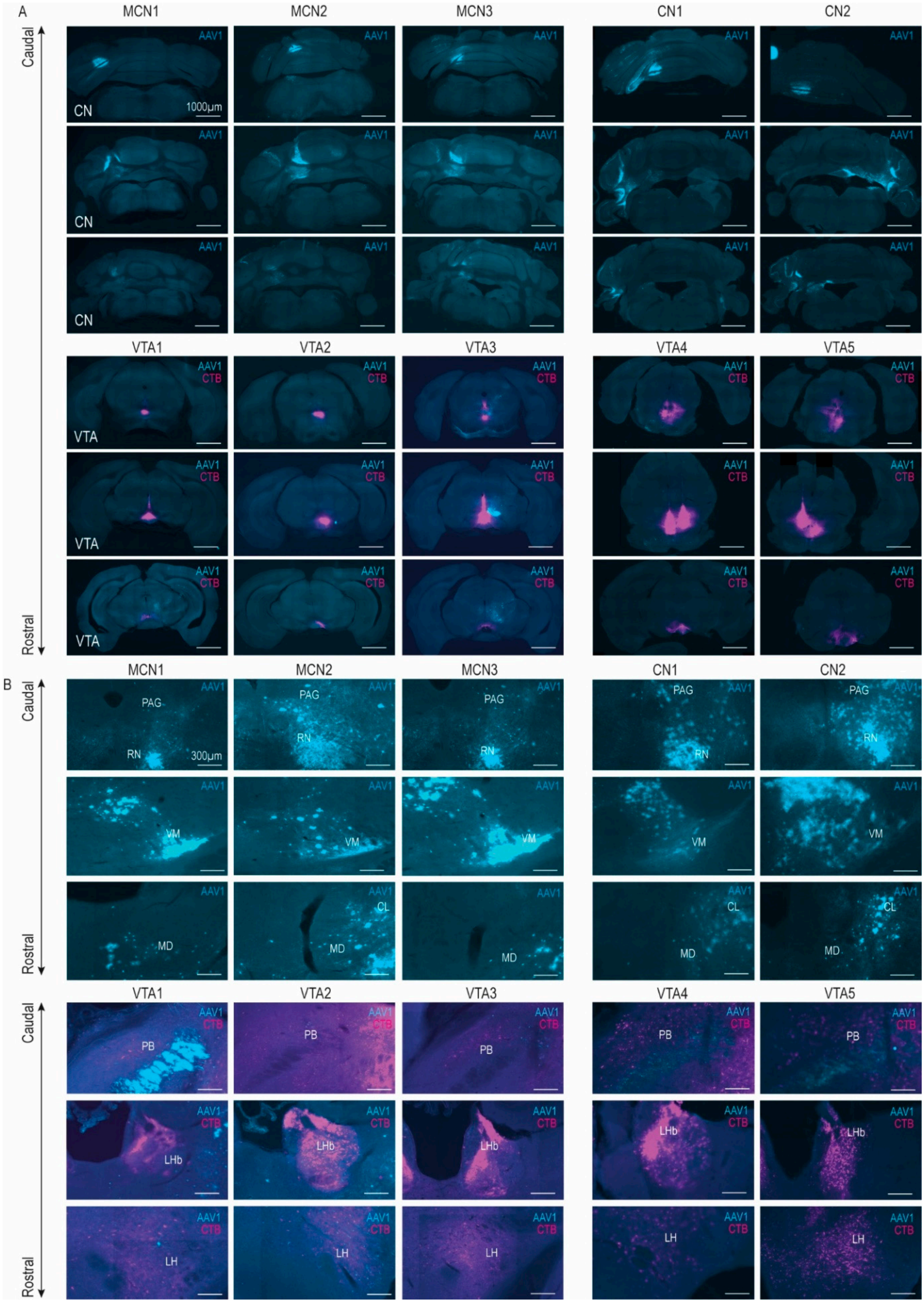

Supplementary Figure S2. Overview of all slices containing injection spots and examples of brain-wide labeling in MCN-VTA mice. (**A**) Overview most caudal (**top**), center (**mid**) and rostral (**bottom**) parts of the injection spot in MCN (first three rows) and VTA (row 4–6) for MCN-only injections (**left**) and MCN-INT-LCN injections (**right**). (**B**) labeling in areas known to receive input from CN (**top**) and provide output to VTA (**bottom**). MCN1–3 are the injections used for

quantification (with accompanying VTA1–3 showing CTB injections in VTA). CN 1–2 are the MCN-INT-LCN injections (with accompanying VTA4–5 indicating CTB injections in VTA). Abbreviations: CL, centrolateral thalamic nucleus; CN, MCN-INT-LCN injections; LH, lateral hypothalamus; LHb, lateral habenula; MD, mediodorsal thalamic nucleus; PAG, periaqueductal gray; PB, parabrachialnucleus; RN, rednucleus; VTA, ventral tegmental area.
